# Objectives and Design of BLEEDS: A Cohort Study to Identify New Risk Factors and Predictors for Major Bleeding during Treatment with Vitamin K Antagonists

**DOI:** 10.1371/journal.pone.0164485

**Published:** 2016-12-09

**Authors:** Nienke van Rein, Willem M. Lijfering, Mettine H. A. Bos, Martien H. Herruer, Helga W. Vermaas, Felix J. M. van der Meer, Pieter H. Reitsma

**Affiliations:** 1 Department of Thrombosis and Hemostasis, Leiden University Medical Center, Leiden, the Netherlands; 2 Einthoven Laboratory for Experimental Vascular Medicine, Leiden University Medical Center, Leiden, the Netherlands; 3 Department of Clinical Epidemiology, Leiden University Medical Center, Leiden, the Netherlands; 4 Atalmedial Medical Diagnostic Centers, Hoofddorp, the Netherlands; 5 Anticoagulation Clinic the Hague, The Hague, the Netherlands; University of Pennsylvania Perelman School of Medicine, UNITED STATES

## Abstract

**Background:**

Risk scores for patients who are at high risk for major bleeding complications during treatment with vitamin K antagonists (VKAs) do not perform that well. BLEEDS was initiated to search for new biomarkers that predict bleeding in these patients.

**Objectives:**

To describe the outline and objectives of BLEEDS and to examine whether the study population is generalizable to other VKA treated populations.

**Methods:**

A cohort was created consisting of all patients starting VKA treatment at three Dutch anticoagulation clinics between January-2012 and July-2014. We stored leftover plasma and DNA following analysis of the INR.

**Results:**

Of 16,706 eligible patients, 16,570 (99%) were included in BLEEDS and plasma was stored from 13,779 patients (83%). Patients had a mean age of 70 years (SD 14), 8713 were male (53%). The most common VKA indications were atrial fibrillation (10,876 patients, 66%) and venous thrombosis (3920 patients, 24%). 326 Major bleeds occurred during 17,613 years of follow-up (incidence rate 1.85/100 person years, 95%CI 1.66–2.06). The risk for major bleeding was highest in the initial three months of VKA treatment and increased when the international normalized ratio increased. These results and characteristics are in concordance with results from other VKA treated populations.

**Conclusion:**

BLEEDS is generalizable to other VKA treated populations and will permit innovative and unbiased research of biomarkers that may predict major bleeding during VKA treatment.

## Introduction

Vitamin K antagonists (VKAs) are used to treat and prevent thromboembolic events [1]. Monitoring of VKA treatment is required because VKAs have a narrow therapeutic window and the dosage depends on inter-individual, but also intra-individual factors [1]. In the Netherlands, patients on VKA treatment are monitored by specialized anticoagulation clinics [2]. The clinics are regionally organized and all patients who live in a certain area are monitored by the same clinic [2]. At these clinics, the international normalized ratios (INRs) are measured on a regular basis, after which a specialized physician determines the VKA dosage and the time interval between INR measurements [2].

Despite this monitoring system, the most common side effects of VKAs remain bleeding complications [1]. Bleeding complications are, depending on the severity, categorized as minor or major bleeding complications. Minor bleedings, such as skin bruises or nosebleeds, occur annually in 6–10% of patients on VKAs and major bleedings, including (fatal) intra-organ bleeds, occur in 1–3% of VKA treated patients per year [2–4]. Risk factors for major bleeding events have been identified and subsequent bleeding risk scores have been developed [5–10]. However, these risk scores do not accurately predict major bleeding (range of C statistics: 0.59–0.69) [11]. Additional biomarkers and genetic variants potentially yield a better accuracy of predicting major bleeding, but information on such predictors is scarce. The goal of the Biomarkers in the Leiden Etiology and Epidemiology of bleeding in vitamin K antagonists Drug users Study (BLEEDS) is to identify novel biomarkers and genetic variants that predict patients at risk for major bleeding events during treatment with VKAs. Here, we delineate the outline of the study. In addition, we provide an overview on classical risk factors for major bleeding to ensure that our population is generalizable to other VKA treated populations.

## Methods

### Study design

BLEEDS is a population based cohort study with longitudinal follow-up in 16,570 patients who started VKA treatment and were recruited from three anticoagulation clinics in the Netherlands.

### Study population

**Fig 1:**
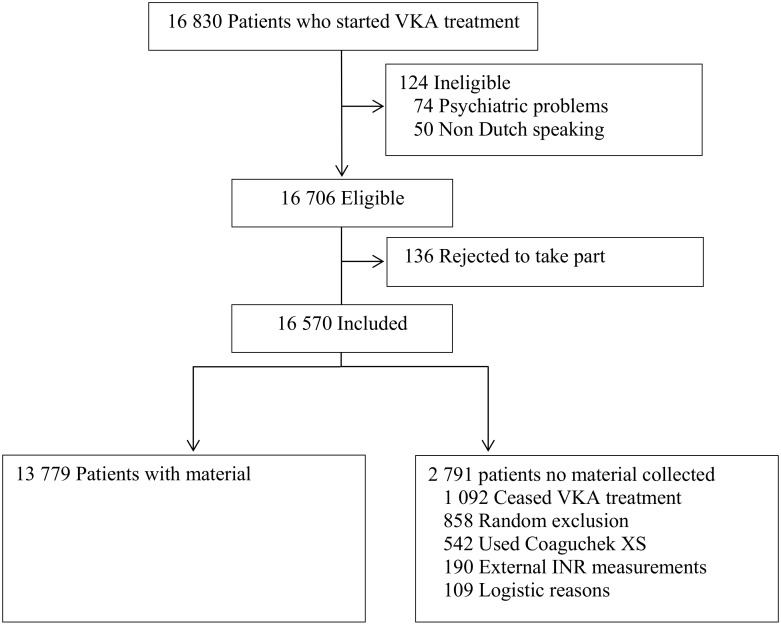
Flow chart of number of individuals included.

Consecutive patients aged 18 years or older who started VKA treatment at one of the three participating anticoagulation clinics in the Netherlands (Leiden, The Hague and Hoofddorp) between January 2012 and July 2014 were eligible (Fig 1). These regional anticoagulation clinics monitor the VKA therapy of those patients living in well-defined geographical areas surrounding Leiden, The Hague and Hoofddorp. Patients were included if the planned treatment duration was at least six weeks, and patients who did not speak Dutch (n = 50) or experienced psychiatric problems (n = 74) were excluded.

Considering an alpha value of 5%, statistical power of 80%, exposure prevalence of 10%, a relative risk of 1.8, an incidence rate of bleeding of 1.8 per year 100 patient years and a mean follow-up of one year, we estimated the necessary sample size at approximately 16,500 patients. All eligible patients received information regarding the study and were included if they did not decline to take part (i.e. an opt-out procedure was followed). This is in accordance with the Dutch law as long as the patient does not have to perform any additional actions for the study and if the privacy of the patients is guaranteed. As more extensively described in the Material collection, left-over plasma was used for the study and patient numbers were encoded to guarantee privacy of the patients. Therefore, the BLEEDS was approved by the medical ethical committee of the Leiden University Medical Center in which an ‘opt-out procedure’ was followed. The included study population consisted of 16,706 eligible patients, of whom 136 opted out (< 1%), resulting in 16,570 included patients.

### Baseline examination and surveillance

When enlisted by the anticoagulation clinic, several patient characteristics were registered, including date of birth, sex, co-medication, indication for VKA treatment, planned duration of VKA treatment and INR target range. To monitor the INR, appointments are made with a frequency of at least every six weeks. The time interval between these measurements depends on the stability of the INR. In case of an unstable INR, the INR will be reassessed more frequently. In case of a stable INR, INR measurements will be performed after a maximum period of six weeks.

To measure the INR, venous blood is drawn into vacuum tubes containing 0.1-volume 0.109 mol/L trisodium citrate as anticoagulant. Blood was centrifuged (10 minutes at 2800 G) within 4 hours of collection, upon which the INR was measured. Another second, less frequently performed method to measure the INR is by using a point-of-care device (CoaguChek XS). At each appointment, a standardized short questionnaire is taken (and electronically stored) by a nurse in order to document changes in co-medication, onset of co-morbidities, the occurrence of bleeding events, or scheduled invasive procedures (e.g. planned surgery or dental extractions).

### Data collection

Patient characteristics were extracted from the computerized patient records of the anticoagulation clinics. Baseline characteristics included sex, age, indication for VKA treatment, type of VKA, INR target range, and co-medications. The study population included 16 185 patients, and because some patients stopped VKA treatment and started again, these patients represented a total of 16,570 treatment periods. There were 8713 male patients (53%) and the mean age was 70 years (standard deviation [SD] 14; Table 1). The most common indications for VKA treatment were atrial fibrillation (10,876 treatment periods, 66%) and venous thrombosis (3920 treatment periods, 24%). Phenprocoumon was used during 12,083 (73%) periods, and approximately half of the patients used antihypertensive medication (8354 patients, 50%) or glucose lowering drugs (8 013 patients, 48%).

**Table 1:** Baseline characteristics.

General characteristics	
Patients	16,185
Treatment periods	16,570
Men	8713 (53)
Age	70 (14)
INR target range	
2.5–3.5	15,509 (93)
3.0–4.0	1061 (7)
Treatment indication	
Atrial fibrillation	10,876 (66)
Venous thrombosis	3920 (24)
Mechanical heart valves	435 (3)
Ischemic heart disease	519 (3)
Vascular disease	412 (3)
Postoperative	125 (1)
Other	348 (2)
Vitamin K antagonist	
Phenprocoumon	12,068 (73)
Acenocoumarol	4481 (27)
Warfarin	18 (0)
Fluindione	3 (0)
Co-Medication	
Anti-platelet drugs	2705 (16)
NSAIDs	1004 (6)
Glucose lowering drugs	8013 (48)
Anti-hypertensive drugs	8354 (50)
Cholesterol lowering drugs	6288 (38)
Digoxin	1767 (11)
Anti-cancer drugs	339 (2)
Opioids	1306 (8)
Methotrexate	155 (1)

### Material collection

For his study, we used patient’s blood and plasma samples that were leftover following INR analyses. The sample collection started three weeks after initiations of VKA therapy and, if applicable, two weeks after termination of low-molecular-weight-heparin (LMWH) treatment. To guarantee the privacy of the patients, technicians who were not involved in the study recoded patient numbers to study numbers. After recoding, patient specific characteristics were concealed and samples were labelled according to study number. The ‘key’ linking patient to study numbers is maintained by a data manager who is not involved in the study. Per patient, a minimum volume of 2.0 ml plasma was collected, which resulted from blood samples of two to three subsequent visits to the anticoagulation clinic. Plasma samples were initially stored at -20°C up to one week. The remaining white blood cells, also encoded with the corresponding study number, were stored for up to one week at 2–8°C, after which DNA was isolated [12]. Plasma and DNA were both long-term stored at -80°C.

Plasma and DNA was collected from 13,779 patients (83%). Material collection failed for 2791 patients because they ceased VKA treatment early (1092 patients), were randomly excluded due to a high workload at the anticoagulation clinic (858 patients), the INR was established by the point-of-care device CoaguChek XS (542 patients), the INR measurement was performed externally (190 patients), and due to logistic complications (109 patients).

### Follow-up and outcome

The follow-up lasted from starting VKA therapy to either the termination of VKA treatment, migration to an area not covered by the three anticoagulation clinics, death, the occurrence of a major bleeding event, or end of the study (31st December 2014), whichever occurred first, resulting in a total follow-up of 17 613 years and a mean follow-up time of 13 months. Patients were followed according to the routine procedures of the anticoagulation clinic. During the appointments, major bleeding events were identified through short interviews that were part of the standard procedures of the anticoagulation clinic. If patients mentioned any bleeding event or hospitalization related to a bleeding event, information was obtained from the hospital, general practitioner or patient to classify the bleeding event as minor or major. Major bleeding events were defined according to the guidelines of the Federation of Dutch Anticoagulation clinics (FNT) by trained anticoagulation clinic physicians, who were not involved in the current study. Bleeding events were classified as major if these were fatal, lead to a blood transfusion or hospital admission, were an intracranial bleeding, objectively diagnosed joint bleed, or a bleeding event in a critical organ [13]. In total, 326 major bleeding events were identified during 17,613 years of follow-up.

## Results and Discussion

BLEEDS provides a large set of patient information and material that will be used to discover new information on risk factors for major bleeding events during VKA treatment. The large number of major bleeding events (n = 326) provides a unique opportunity to perform subgroup analyses and study relatively rare risk factors.

Previously, three studies have been performed in which plasma and DNA were collected to discover new risk factors for major bleeding during VKA treatment [14–16]. However, in all of these studies, only a subgroup of the total VKA-treated patients was included. The first study excluded patients who died or became mentally disabled by the bleeding event and included only 22% of all patients who experienced a major bleeding complication in the final analyses [16]. This makes the results susceptible to survivor bias [17]. The second study’s inclusion criterion was a time in therapeutic range (TTR) of 100% [15], while the third study included only 75% of the warfarin-treated patients. Furthermore, the patients were followed up to 5.5 years [14], which may have diluted the results. By including only subsets of patients on VKA therapy, results can be biased and cannot be extrapolated to all VKA-treated patients [16, 17]. In BLEEDS, follow-up was short which creates the possibility to study risk factors that predict the short term risk for major bleeding. In addition, we have included 99% of the eligible patients treated with VKAs, thereby providing a strong case that our population represents real world patients.

Further support for this stems from the observation that the incidence rate of the major bleeding events (1.85 per 100 patient-years, 95% CI 1.66–2.06; see Table 2) compares well with major bleeding rates of other population-based studies [2, 18, 19]. The major bleeding complications observed in our study also concur with previous findings, given that intracranial bleedings were the most common fatal major bleeding complications (37, 54% of all fatal major bleeding events), while the non-fatal bleedings mostly resulted from digestive (106, 41%) and intracranial (40, 15%; see Table 3) bleedings [2, 18, 19].

**Table 2:** Incidence rates of bleeding events stratified by clinical characteristics.

	No. ofevents	Patient time (years)	Events/100 patient-years(95% CI)
Total	326	17,613	1.85 (1.66–2.06)
Sex			
Male	184	9224	1.99 (1.72–2.30)
Female	142	8387	1.69 (1.43–1.99)
INR target range			
2.5–3.5	306	16,454	1.86 (1.66–2.08)
3.0–4.0	20	1157	1.73 (1.09–2.62)
Vitamin K antagonist			
Phenprocoumon	262	13,278	1.97 (1.75–2.22)
Acenocoumarol	64	4333	1.48 (1.15–1.87)
Indication			
Atrial fibrillation	241	13,162	1.83 (1.61–2.07)
Venous thrombosis	53	2702	1.96 (1.48–2.55)
Mechanical heart valves	4	351	1.14 (0.36–2.75)
Ischemic hearts disease	7	555	1.26 (0.55–2.50)
Vascular	9	433	2.08 (1.01–3.81)
Postoperative	3	105	2.86 (0.72–7.78)
Other	9	384	2.34 (1.14–4.30)

**Table 3:** Number of bleeding events stratified by area.

	Fatal	Non-fatal
Total	68	260
Gastrointestinal	12 (18)	106 (41)
Intracranial	37 (54)	40 (15)
Muscle	0	13 (5)
Joint	0	17 (7)
Epistaxis	0	16 (6)
Respiratory	3 (4)	9 (3)
Urinary tract	1 (1)	16 (6)
Ocular	0	1 (0)
Skin	0	8 (3)
Circulatory	9 (13)	6 (2)
Retroperitoneal	0	1 (0)
Other	6 (9)	27 (10)

**Fig 2:**
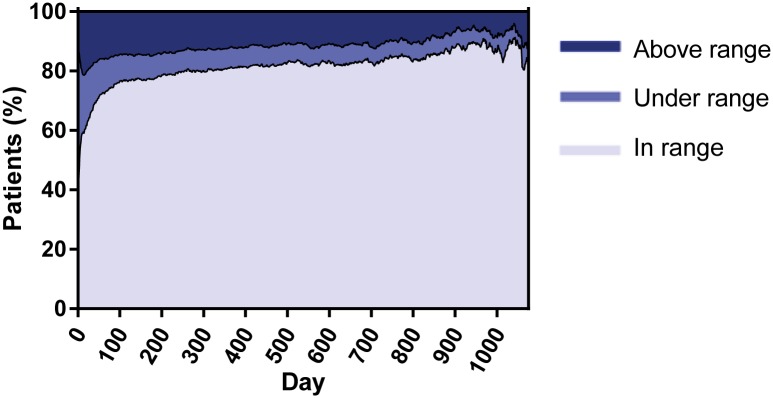
Time in therapeutic range per day after starting VKA treatment.

To further analyze if BLEEDS agrees with other VKA treated populations in terms of predictors for bleeding, we decided to calculate the TTR by linear interpolation as described by Rosendaal *et al*. [20] Previous studies have shown that high TTRs are associated with lower bleeding rates as compared with low TTRs. The lowest TTR was observed shortly after the initiation of VKA treatment, which increased to approximately 80% and stabilized until the end of follow-up (Fig 2), which in agreement with previous findings. This pattern is as expected based on other population based studies, although it should be mentioned that a TTR of 80% is within the upper range of normal for Western European anticoagulation clinics that on average achieve a TTR of 70% [21].

**Fig 3:**
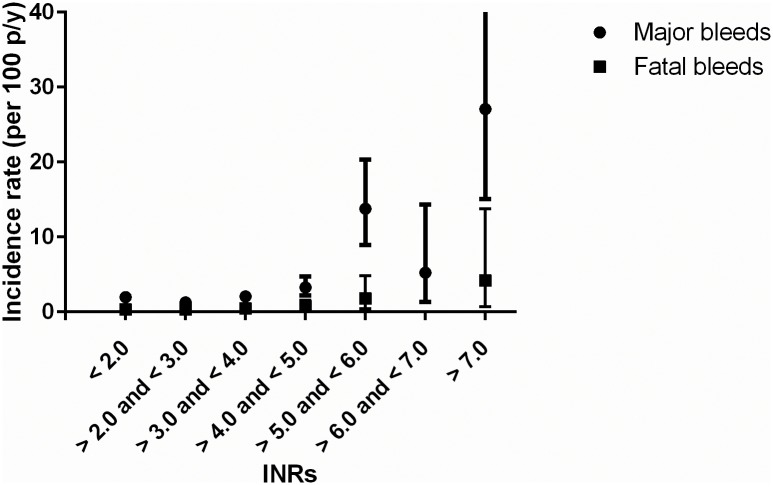
Incidence rates of bleeding events stratified by age.

**Fig 4:**
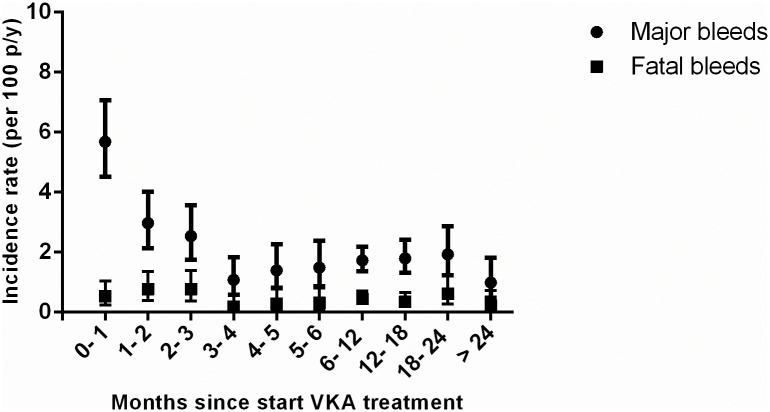
Incidence rates of bleeding events stratified by time since start of VKA treatment.

**Fig 5:**
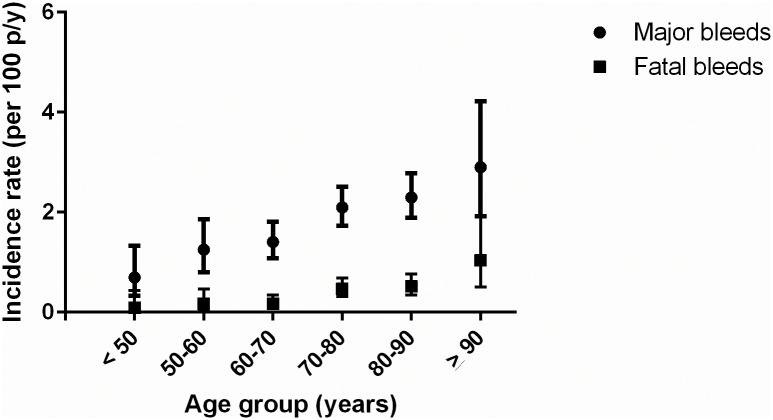
Incidence rates of bleeding events stratified by INR.

Further TTR assessment revealed that a low TTR was dose-dependently associated with increased bleeding rates (see Table 4) [20, 22, 23]. Additional analyses confirmed that, similar to other population based cohorts, in BLEEDS the bleeding rates i) increased with age (Fig 3) [2, 10, 18, 24–26], ii) were highest shortly after initiation of VKA treatment and became stable after four months of VKA treatment (Fig 4) [18, 25, 27, 28], iii) and increased after a high INR (Fig 5) [18, 26]. The number of fatal bleeding events was stable from start of therapy, but increased with age and INR (Figs 3 to 5). Results of the subgroup analyses (atrial fibrillation patients, venous thrombosis patients, and patients with an INR target range between 2.5 and 3.5) showed similar results as compared with the main analyses (see S1–S4 Figs, S1 and S2 Tables).

**Table 4:** Association of TTR with bleeding events.

	No. of events	Patient years	Events/100 patient-years (95% CI)
Time in range			
< 35%	63	448	13.02 (10.09–16.54)
≥ 35% and < 50%	33	1 145	2.88 (2.02–4.00)
≥ 50% and < 60%	35	1 538	2.28 (1.61–3.13)
≥ 60% and < 70%	48	2 212	2.17 (1.62–2.85)
≥ 70% and < 80%	40	2 821	1.42 (1.03–1.91)
≥ 80% and < 90%	37	3 059	1.21 (0.86–1.65)
≥ 90%	62	5 361	1.16 (0.89–1.47)

Two characteristics of the study deserve a further comment. Material (plasma and DNA) of all patients was collected from the third week after initiating VKA therapy and, if applicable, two weeks after termination of LMWH therapy. As such, we did not collect material of 32 patients who experienced a major bleeding complication before blood could be collected of which 10 patients died and 22 ceased VKA treatment. As a possible consequence, risk estimates may dilute because the patients with the strongest risk factors ‘dropped out’ of the study, which may disfavor the study of patients with the strongest and thus shortest term risk factors. Second, the low INR target range is 2.5–3.5 in the Netherlands as decided by the Federation of Dutch Anticoagulation Clinics, while it is 2.0–3.0 in other countries. This may result in slightly higher major bleeding rates in these patients, but may also favor the detection of risk factors for major bleeding complications.

In this study, 99% of the eligible patients were included which results in a unique, unselected study population. The number of major bleeding events is relatively high, which creates the possibility to perform subgroup analyses and study whether protein levels are dose dependently associated with major bleeding events. In addition, the availability of stored biological specimens (citrated plasma and DNA) will allow us to uncover new risk factors for major bleeding complications during VKA treatment, with the goal of discovering new predictors for VKA-treated patients at high risk for major bleeding events. We would like to emphasize that results of BLEEDS with respect to classical risk factors for major bleeding are similar to other cohorts of patients who received VKAs for all long term indications, which indicates that this population is generalizable to other populations. In summary, the BLEEDS will permit innovative and unbiased research of multiple exposures for major bleeding events and will assist in the prevention of major bleeding events in patients treated with VKAs.

## Supporting Information

S1 Table Incidence rates of bleeding events stratified by clinical characteristics (atrial fibrillation patients, venous thrombosis patients, patients with a low target range). (DOCX)

S2 Table Association of TTR with bleeding events stratified by clinical characteristics (atrial fibrillation patients, venous thrombosis patients, patients with a low target range). (DOCX)

S1 Fig Time in therapeutic range per day after starting VKA treatment presented per sub group (atrial fibrillation patients, venous thrombosis patients, patients with a low target range). (DOCX)

S2 Fig Incidence rates of bleeding events stratified by age presented per sub group (atrial fibrillation patients, venous thrombosis patients, patients with a low target range). (DOCX)

S3 Fig Incidence rates of bleeding events stratified by time since start of VKA treatment presented per sub group (atrial fibrillation patients, venous thrombosis patients, patients with a low target range). (DOCX)

S4 Fig incidence rates of bleeding events stratified by INR, presented per sub group (atrial fibrillation patients, venous thrombosis patients, patients with a low target range). (DOCX)
