# Supplementary material for: Objectives and Design of BLEEDS: A Cohort Study to Identify New Risk Factors and Predictors for Major Bleeding during Treatment with Vitamin K Antagonists
Source: PLoS One. 2016 Dec 9;11(12):e0164485. doi: 10.1371/journal.pone.0164485 (PMC5147785; doi:10.1371/journal.pone.0164485)
Supplement: S1 Table — (DOCX) [file pone.0164485.s001.docx]

|  |  |  |  |  | AF patients | VT patients | Low target range |
| --- | --- | --- | --- | --- | --- | --- | --- |
|  |  |  |  | Events/100 | Events/100 | Events/100 | Events/100 |
|  |  | No. of  events | Patient time (years) | patient-years  (95% CI) | patient-years  (95% CI) | patient-years  (95% CI) | patient-years  (95% CI) |
| Total | | 326 | 17 613 | 1.85 (1.66-2.06) | 1.83 (1.61-2.07) | 1.96 (1.48-2.55) | 1.86 (1.66-2.08) |
| Sex | |  |  |  |  |  |  |
|  | Male | 184 | 9 224 | 1.99 (1.72-2.30) | 1.99 (1.68-2.35) | 2.12 (1.45-3.01) | 2.02 (1.73-2.34) |
|  | Female | 142 | 8 387 | 1.69 (1.43-1.99) | 1.66 (1.37-2.00) | 1.80 (1.18-2.63) | 1.69 (1.43-2.00) |
| INR target range | |  |  |  |  |  |  |
|  | 2.5-3.5 | 306 | 16 454 | 1.86 (1.66-2.08) | 1.83 (1.61-2.08) | 1.98 (1.50-2.57) | NA |
|  | 3.0-4.0 | 20 | 1 157 | 1.73 (1.09-2.62) | 1.70 (0.08-8.36) | * | NA |
| Vitamin K antagonist | |  |  |  |  |  |  |
|  | Phenprocoumon | 262 | 13 278 | 1.97 (1.75-2.22) | 1.92 (1.67-2.21) | 2.10 (1.54-2.80) | 1.96 (1.73-2.22) |
|  | Acenocoumarol | 64 | 4 333 | 1.48 (1.15-1.87) | 1.53 (1.14-2.02) | 1.54 (0.78-2.74) | 1.53 (1.18-1.96) |
| Indication | |  |  |  |  |  |  |
|  | Atrial fibrillation | 241 | 13 162 | 1.83 (1.61-2.07) | NA | NA | 1.83 (1.61-2.08) |
|  | Venous thrombosis | 53 | 2 702 | 1.96 (1.48-2.55) | NA | NA | 1.98 (1.50-2.57) |
|  | Mechanical heart valves | 4 | 351 | 1.14 (0.36-2.75) | NA | NA | 1.53 (0.49-3.68) |
|  | Ischemic heart disease | 7 | 555 | 1.26 (0.55-2.50) | NA | NA | 1.13 (0.19-3.73) |
|  | Vascular | 9 | 433 | 2.08 (1.01-3.81) | NA | NA | * |
|  | Postoperative | 3 | 105 | 2.86 (0.72-7.78) | NA | NA | 3.13 (0.79-8.51) |
|  | Other | 9 | 384 | 2.34 (1.14-4.30) | NA | NA | 2.90 (0.92-6.99) |
| * No events occurred in this subgroup | | | | | | | |
|  | | | | | | | |
