# Supplementary material for: Objectives and Design of BLEEDS: A Cohort Study to Identify New Risk Factors and Predictors for Major Bleeding during Treatment with Vitamin K Antagonists
Source: PLoS One. 2016 Dec 9;11(12):e0164485. doi: 10.1371/journal.pone.0164485 (PMC5147785; doi:10.1371/journal.pone.0164485)
Supplement: S2 Table — (DOCX) [file pone.0164485.s002.docx]

|  |  |  |  |  |  |  |  |
| --- | --- | --- | --- | --- | --- | --- | --- |
|  |  |  |  | Events/100 patient-years (95% CI) | | | |
|  |  | No. of | Patient |  |  |  |  |
|  |  | events | years | All | AF patients | VT patients | Low target range |
| Time in range | |  |  |  |  |  |  |
| < 35% | | 63 | 448 | 13.02 (10.09-16.54) | 14.33 (10.65-18.89) | 16.92 (8.90-29.41) | 14.59 (11.26-18.62) |
| > 35% and < 50% | | 33 | 1 145 | 2.88 (2.02-4.00) | 3.38 (2.27-4.85) | 0.59 (0.03-2.90) | 3.06 (2.12-4.30) |
| > 50% and < 60% | | 35 | 1 538 | 2.28 (1.61-3.13) | 2.33 (1.55-3.36) | 2.54 (1.03-5.29) | 2.35 (1.64-3.26) |
| > 60% and < 70% | | 48 | 2 212 | 2.17 (1.62-2.85) | 0.84 (0.58-1.17) | 0.98 (0.40-2.04) | 2.16 (1.59-2.88) |
| > 70% and < 80% | | 40 | 2 821 | 1.42 (1.03-1.91) | 0.65 (0.45-0.91) | 1.35 (0.73-2.30) | 1.36 (0.97-1.86) |
| > 80% and < 90% | | 37 | 3 059 | 1.21 (0.86-1.65) | 0.55 (0.38-0.78) | 0.43 (0.16-0.96) | 1.18 (0.83-1.62) |
| > 90% | | 62 | 5 361 | 1.16 (0.89-1.47) | 0.42 (0.31-0.55) | 0.51 (0.27-0.88) | 1.21 (0.93-1.54) |
