# Supplementary figures and images for: Objectives and Design of BLEEDS: A Cohort Study to Identify New Risk Factors and Predictors for Major Bleeding during Treatment with Vitamin K Antagonists
Source: PLoS One. 2016 Dec 9;11(12):e0164485. doi: 10.1371/journal.pone.0164485 (PMC5147785; doi:10.1371/journal.pone.0164485)

All AF patients


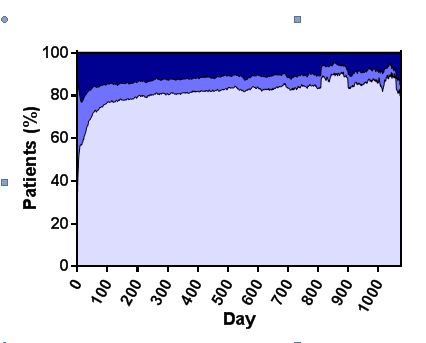

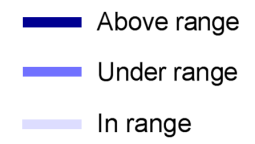

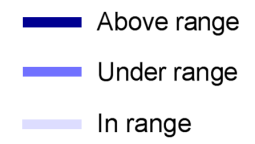

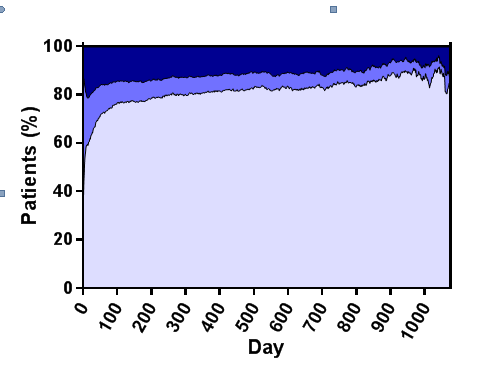


VT patients Low target range


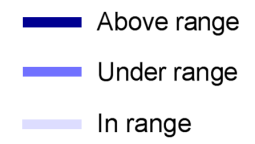


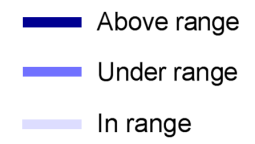

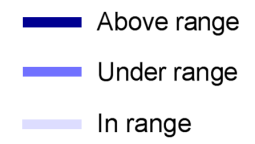

Supplement: S1 Fig — (DOCX) [file pone.0164485.s003.docx]
